# Jackknife-based gene-gene interactiontests for untyped SNPs

**DOI:** 10.1186/s12863-015-0225-9

**Published:** 2015-07-18

**Authors:** Minsun Song

**Affiliations:** Division of Cancer Epidemiology and Genetics, National Cancer Institute, National Institutes of Health, 9609 Medical Center Drive, Rockville, MD USA

**Keywords:** Jackknife-based testing framework, Untyped SNP, Imputation-based testing, Gene-gene interaction

## Abstract

**Background:**

Testing gene-gene interaction in genome-wide association studies generally yields lower power than testing marginal association. Meta-analysis that combines different genotyping platforms is one method used to increase power when assessing gene-gene interactions, which requires a test for interaction on untyped SNPs. However, to date, formal statistical tests for gene-gene interaction on untyped SNPs have not been thoroughly addressed. The key concern for gene-gene interaction testing on untyped SNPs located on different chromosomes is that the pair of genes might not be independent and the current generation of imputation methods provides imputed genotypes at the marginal accuracy.

**Results:**

In this study we address this challenge and describe a novel method for testing gene-gene interaction on marginally imputed values of untyped SNPs. We show that our novel Wald-type test statistics for interactions with and without constraints in the interaction parameters follow the asymptotic distributions which are the same as those of the corresponding tests for typed SNPs. Through simulations, we show that the proposed tests properly control type I error and are more powerful than the extension of the classical dosage method to interaction tests. The increase in power results from a proper correction for the uncertainty in imputation through the variance estimator using the jackknife, one of resampling techniques. We apply the method to detect interactions between SNPs on chromosomes 5 and 15 on lung cancer data. The inclusion of the results at the untyped SNPs provides a much more detailed information at the regions of interest.

**Conclusions:**

As demonstrated by the simulation studies and real data analysis, our approaches outperform the application of traditional dosage method to detection of gene-gene interaction in terms of power while providing control of the type I error.

**Electronic supplementary material:**

The online version of this article (doi:10.1186/s12863-015-0225-9) contains supplementary material, which is available to authorized users.

## Background

Genome-wide association studies (GWAS) have been an important tool to discover single nucleotide polymorphisms (SNPs), which are associated with disease [1–3]. However, the development of a disease prediction model based on established susceptibility SNPs from GWAS has been less successful for complex diseases. One of the main reasons in such a failure is that they do not take into account gene-gene interactions [4]. Past efforts to analyze interactions between SNPs have been limited, since discovery and replication of these findings is difficult in GWAS. Since the effect size of the gene-gene interaction is small, the sample size from one study might not be large enough to detect the interaction between genes. One way to increase power for detecting gene-gene interaction is by performing a meta-analysis for the gene-gene interaction that combines disparate datasets from studies where genotyping platforms differ in terms of SNP sets [5]. Therefore, not only there is a need for better gene-gene interaction test, but also for tests that are specifically developed for interactions between untyped SNPs that are not included in the genotyping platform in a meta-analytic approach.

In the context of single-locus analysis, several approaches have been proposed for testing associations for untyped SNPs. One is the full likelihood approach where the observed-data likelihood is derived by integrating the study data and external reference data over the possible haplotype configuration, and imputing and testing are performed simultaneously [6]. Such a full likelihood-based approach dealing with both imputing and testing under a unified framework will lead to efficient estimators of the association parameters. Also, the corresponding variance estimators will properly take into account uncertainties of imputed values. However, even if the principle of maximum likelihood is fairly simple, obtaining the actual solution can be computationally intensive given that the likelihood involves the sum over the pairs of haplotypes that are compatible with the genotypes. Numerical optimization would not be tractable when a complex model is used for inferring genotypes at the untyped SNPs.

Another type of association analysis for untyped SNPs is the two-stage procedure where in the first stage, imputed genotypes for untyped SNPs are obtained and at the second stage, the imputed genotypes are used in a downstream analysis. This strategy could be less efficient and satisfactory compared to approaches that use a proper joint modeling [7]. However, from an operational point of view, the two-stage procedure has its own advantage. There have been many powerful approaches including softwares for the first step which allow users to impute missing genotypes in a sophisticated and convenient way [8–14]. In the two-stage procedure, it is a standard to replace the value of missing genotype by dosage [15], which is the expected genotype count for each individual, and analyze the data by the standard association analysis. The Wald test based on the logistic regression model where disease status is response and dosages are covariates, is valid. However, for the purpose of detecting interactions, it has been shown that for typed SNPs, under the gene-gene independence, the test under the logistic regression model based on case-control data is less powerful than test for cases only, which is based on genotype distribution conditional on cases [16]. Unfortunately, for untyped SNPs, the Wald test based on genotype distribution (rather than disease distribution conditioned on genotype) would be conservative when dosage is treated as observed data (e.g., de Bakker *et al.* [17]). This is due to overestimation of the variance.

The central aim of the paper is to develop powerful tests for interaction on imputed genotypes at untyped SNPs in the second stage of the two-stage strategy which has practical advantage. We do not address the issue of imputation itself. Rather, we address how to perform interaction tests on imputed values given from the imputation approaches and issue of how the performance of proposed tests depends on linkage disequilibrium (LD) among SNPs. Intuitively, jointly imputed values are required for testing gene-gene interactions. However, to our knowledge, most imputation analyses have imputed genotypes at missing SNPs marginally and formal statistical tests for gene-gene interactions which could be performed on marginally imputed genotypes have not been developed. In this article, we propose statistical tests which could be applied on marginally imputed data from external imputation algorithms, a Wald-type test (WTT) and a Wald-type test with constraints (WTT_*c*_), that are constructed under the null. We consider a “no interaction” model for two unlinked loci which is defined in Song and Nicolae [16], multiplicative penetrances, as the null. The Wald-type statistics require the null covariance matrix properly capturing the uncertainty of imputation. To accomplish this, we use the jackknife, one of the resampling methods. Specifically, WTT_*c*_ is an extension of inequality constrained penetrance test, the approach of Song and Nicolae [16], which has been shown to be more powerful than classical tests of gene-gene interactions by restricting parameter space with inequalities. We investigate the type I error and power of our tests by conducting extensive simulation studies, and apply the method to the real data example.

## Methods

### Test statistics

We consider SNP-SNP interaction for gene-gene interaction analysis. Suppose we have the untyped markers *G* and *H*, and we assume that they are in linkage equilibrium (LE) in the general population. Let *a* and *A* be the two alleles at the marker *G* and *b* and *B* the two alleles at the marker *H*. Genotypes at marker *G* (*H*), *aa*,*Aa*, and *AA* (*bb*,*Bb*, and *BB*), can be indexed by 0, 1, and 2, respectively. Suppose that we have a case-only study with a total of *n* samples *y*_1_,…,*y*_*n*_. For the *i*th sample, let *y*_*i*,*obs*_ and *y*_*i*,*miss*_ be the observed data and missing data of *y*_*i*_, respectively. Therefore, for the untyped markers *G* and *H*, *y*_*i*,*miss*_=(*y*_*i*,*G*_,*y*_*i*,*H*_) and *y*_*i*,*obs*_ is the observed genotype data which are in LD with markers *G* and *H* for subject *i*. We use ***p***_*i*_=(*p*_*i*,00_,*p*_*i*,01_,…,*p*_*i*,21_,*p*_*i*,22_) to denote a conditional distribution for the joint genotype of markers *G* and *H* for the *i*th sample (e.g., *p*_*i*,00_=*P*((*y*_*i*,*G*_,*y*_*i*,*H*_)=(*aa*,*bb*)|*y*_*obs*_,***η***)), where ***η*** is the parameter associated with an imputation model.

We consider “no interaction” of Song and Nicolae [16] (i.e., multiplicative penetrances) as the null hypothesis for testing gene-gene interaction. “No interaction” of Song and Nicolae [16] is equivalent to ***β***=**0** where local odds ratios ***β***=(*β*_00_,*β*_01_,*β*_10_,*β*_11_) are defined as follows,

(1) $$ \beta_{lm}=\log\left(\frac{p_{l,m} p_{l+1,m+1}}{p_{l,m+1}p_{l+1,m}}\right), l,m=0,1  $$

where *p*_*lm*_=*P*(*y*_*G*_=*l*,*y*_*H*_=*m*) among cases. These can be estimated as

(2) $$ \hat{\beta}_{lm}=\log\left(\frac{n_{l,m}n_{l+1,m+1}}{n_{l,m+1}n_{l+1,m}}\right),  $$

where *n*_*l*,*m*_ is the count of joint genotypes when markers *G* and *H* take genotypes *l* and *m* in *n* cases, respectively. Note that $n_{l,m}=\sum _{i=1}^{n} I(y_{i,G}=l, y_{i,H}=m)$.

The form of the estimator (2) suggests how it can be modified for untyped SNPs. For untyped SNPs, $\hat {\boldsymbol {\beta }}^{u}=T(E(N_{l,m}|y_{\textit {obs}}, \boldsymbol {\eta }))$, *l*,*m*=0,1,2 for function $T: \mathbb {R}^{9} \rightarrow \mathbb {R}^{4}$, where *N*_*l*,*m*_ is a random variable with a distribution *F*(*n*_*l*,*m*_)∈{*F*_***η***_(*n*_*l*,*m*_);***η***∈*Ω*}. When the markers *G* and *H* are typed, *n*_*l*,*m*_ is the observation of random variable *N*_*l*,*m*_. One can replace *n*_*l*,*m*_ in (2) with

$$\begin{array}{@{}rcl@{}} E\left(N_{l,m}|y_{obs}, \boldsymbol{\eta}\right)& = & E\left(\sum\limits_{i=1}^{n} I\left(y_{i,G}=l, y_{i,H}=m\right)|y_{obs}, \boldsymbol{\eta}\right)\\ &=& \sum\limits_{i=1}^{n} E\left(I\left(y_{i,G}=l, y_{i,H}=m\right)|y_{obs}, \boldsymbol{\eta}\right)\\ &=& \sum\limits_{i=1}^{n} p_{i, lm}. \end{array} $$

Under the null hypothesis of no interaction,

$$ \begin{aligned} \hat{p}_{i,lm,0}&:=\! P\left(y_{i,G}\,=\,l, y_{i,H}\,=\,m|y_{obs}, \hat{\boldsymbol{\eta}}_{0}\right)\,=\, P\left(y_{i,G}=l|y_{obs}, \hat{\boldsymbol{\eta}}_{0}\right)\\ &\quad\times P\left(y_{i,H}=m|y_{obs},\hat{\boldsymbol{\eta}}_{0}\right) \end{aligned} $$

where ***η***_0_ is evaluated under the null hypothesis “multiplicative penetrances”. Thus, we could obtain the imputed joint genotypes by taking a product of the marginally imputed genotypes from most of imputation methods. The log local odds ratios for untyped SNPs evaluated under the null can be written as

(3) $$ \hat{\beta}_{lm}^{0u}=\log\left(\frac{E\left[N_{l,m}|y_{obs}, \hat{\boldsymbol{\eta}}_{0}\right]E\left[N_{l+1,m+1}|y_{obs}, \hat{\boldsymbol{\eta}}_{0}\right]}{E\left[N_{l,m+1}|y_{obs},\hat{\boldsymbol{\eta}}_{0}\right] E\left[N_{l+1,m}|y_{obs},\hat{\boldsymbol{\eta}}_{0}\right]}\right)  $$

where $E\left [N_{l,m}|y_{\textit {obs}},\hat {\boldsymbol {\eta }}_{0}\right ]=\sum _{i=1}^{n} \hat {p}_{i,lm,0}$. $E\left [N_{l,m}|y_{\textit {obs}},\hat {\boldsymbol {\eta }}_{0}\right ]$ can be considered as a special case of dosage under the null hypothesis : ***β***=**0**.

The Wald test will be valid as long as the variance estimator is consistent (so the Wald test statistic follows the chi-square distribution asymptotically). Dealing with the variance is often much easier than manipulating the likelihood functions. Thus, we consider a form of Wald test using a consistent variance estimator for $\hat {\boldsymbol {\beta }}$. When both markers are observed, the Wald test statistic for interaction of two markers is

(4) $$ \hat{\boldsymbol{\beta}}^{T}\hat{\boldsymbol{V}}^{-1}\hat{\boldsymbol{\beta}}  $$

where $\hat {\boldsymbol {V}}$ is the consistent estimator for variance of $\hat {\boldsymbol {\beta }}$ and

$$\textbf{V}=\frac{1}{n} \left(\begin{array}{cccc} \frac{1}{p_{00}}+\frac{1}{p_{01}}+\frac{1}{p_{10}}+\frac{1}{p_{11}} & -\frac{1}{p_{01}}-\frac{1}{p_{11}}& -\frac{1}{p_{10}}-\frac{1}{p_{11}}& \frac{1}{p_{11}} \\ & \frac{1}{p_{01}}+\frac{1}{p_{02}}+\frac{1}{p_{11}}+\frac{1}{p_{12}} & \frac{1}{p_{11}}& -\frac{1}{p_{11}}-\frac{1}{p_{12}}\\ & & \frac{1}{p_{10}}+\frac{1}{p_{11}}+\frac{1}{p_{20}}+\frac{1}{p_{21}}& -\frac{1}{p_{11}}-\frac{1}{p_{21}}\\ & & & \frac{1}{p_{11}}+\frac{1}{p_{12}}+\frac{1}{p_{21}}+\frac{1}{p_{22}} \end{array}\right). $$

The test for interaction on untyped SNPs can be similarly defined in the form of Wald-Type test (WTT)

(5) $$ \text{WTT}=\left(\hat{\boldsymbol{\beta}}^{0u}\right)^{T}\left\{\hat{\boldsymbol{V}}^{0u}\right\}^{-1}\hat{\boldsymbol{\beta}}^{0u}  $$

where $\hat {\boldsymbol {V}}^{0u}$ is the consistent estimator for variance of $\hat {\boldsymbol {\beta }}^{0u}$. The test statistic (5) has an approximate ${\chi ^{2}_{4}}$ distribution.

Inequality constrained penetrance test (ICPT) [16], the likelihood ratio test (LRT) with restrictions on interaction parameters under the alternative, has been shown to be a powerful approach for identifying gene-gene interactions in the presence of directional constraints. The imposed constraints are *β*_*lm*_ for *l*,*m*=0,1 are all non-negative or all non-positive, which hold in the models presented in Table 1. We expand ICPT to untyped SNPs by approximating the LRT statistic by a Wald-like test statistic form using a Taylor expansion. It could be shown that LRT statistic is approximated by a quadratic form [18]. Therefore ICPT for untyped SNPs can be written as Wald-type test with constraints (WTT_*c*_)

(6) $$ {\small{ \begin{aligned} \text{WTT}_{c}&\,=\,\left(\hat{\boldsymbol{\beta}}^{0u}\right)^{T}\left\{\hat{\boldsymbol{V}}^{0u}\right\}^{-1} \hat{\boldsymbol{\beta}}^{0u}-\min_{\boldsymbol{\beta}^{*}\in\Theta_{1}} \left(\hat{\boldsymbol{\beta}}^{0u}- \boldsymbol{\beta}^{*}\right)^{T} \left\{\hat{\boldsymbol{V}}^{0u}\right\}^{-1} \\ &\qquad\times\left(\hat{\boldsymbol{\beta}}^{0u}-\boldsymbol{\beta}^{*}\right) \end{aligned}}}  $$

**Table 1:** Penetrance tables for two disease loci (*g*<*f*)

D ∪D			R ∪R			D ∪R		
*g*	*f*	*f*	*g*	*g*	*f*	*g*	*g*	*f*
*f*	*f*	*f*	*g*	*g*	*f*	*f*	*f*	*f*
*f*	*f*	*f*	*f*	*f*	*f*	*f*	*f*	*f*
R ∩D			R ∩R			D ∩D		
*g*	*g*	*g*	*g*	*g*	*g*	*g*	*g*	*g*
*g*	*g*	*g*	*g*	*g*	*g*	*g*	*f*	*f*
*g*	*f*	*f*	*g*	*g*	*f*	*g*	*f*	*f*

D ∪D, the union of dominant and dominant; R ∪R, the union of recessive and recessive; D ∪R, the union of dominant and recessive; R ∩D, the intersection of recessive and dominant; R ∩R, the intersection of recessive and recessive; D ∩D, the intersection of dominant and dominant [This table is adapted from Table 1 of Song and Nicolae [16]]

where *Θ*_1_ is an inequality-constrained space of parameter ***β***. With a consistent variance estimate of ***V***^0*u*^ and appropriate weights obtained through the proper variance estimator (see Additional file 1), the test (6) follows a chi-bar distribution with 1-4 degrees of freedom asymptotically under the null hypothesis (i.e., $P(\text {WTT}_{c} \ge t)\approx \sum _{l=1}^{4}w_{l} P(\chi ^{2}_{{l}} \ge t)$).

### Variance estimation

The $\hat {\boldsymbol {\beta }}^{0u}$ can be viewed as a function of expected counts under the null, which is dosage under the null. The Wald test based on the distribution of disease status conditioned on dosage is valid when the unobserved genotype on untyped SNP is replaced by dosage (e.g., the logistic regression model where the response variable is disease status and the input variable is dosage). However, the Wald test constructed on genotype distribution conditioned on disease status is not valid anymore if one simply replaces the unmeasured genotype on untyped SNP as dosage. The latter case includes a Wald test where null is ***β***=**0**, constructed on the joint distribution of genotypes conditioned on cases. In the case, one needs to take into account the imputation in the variance. Nevertheless, it is often challenging to derive the asymptotic variance accounting for the uncertainty of imputed values under the complex model of inferring genotypes at untyped SNPs. To overcome the difficulty, one approach of obtaining the consistent variance estimator is to use the jackknife. The basic idea behind the jackknife lies in systematically recomputing the statistic leaving out one observation at a time from the sample set when samples are independent and identically distributed. In our case, in principle, we need to repeat both imputation and computation of test statistics although it is tempting to apply a resampling method on the imputed data. However, for large data and computationally intensive imputation approaches, repetition of imputation is not desirable.

To reduce the computational time, we can consider the imputation approach for which imputation for one study individual does not depend on others in study data (i.e. $\hat {\boldsymbol {p}}_{i}$ does not depend on {*y*_*j*,*obs*_,*j*≠*i*}). By obtaining $\hat {\boldsymbol {\eta }}$ only from an external reference panel, such as the HapMap [19] rather than together with the study data, we can impute the unobserved genotypes in study individual independent of the other individuals on whom imputation will be performed. In this case, $\hat {\boldsymbol {p}}_{i}$ does not change in the “new” dataset in resampling. Therefore, resampling can be applied directly on imputed values from such imputation approaches (e.g., IMPUTE [12]).

Even though the resampling method is a useful tool for estimating the sampling variability of a statistic from complex procedures, the disadvantage of resampling methods is the excessive computation. In terms of the computational challenge, several important points for using the jackknife over the bootstrap in our proposed framework emerge as follows. First, while many existing imputation methods use the available information only from the reference panel to learn about the parameters in the imputation model, IMPUTE2 [14], one of the popular imputation approaches, uses all of the available information from study dataset as welll as the reference panel. In the case, when we make a “new” set of data from resampling, in principal, one needs to repeat the imputation. However, unlike the bootstrap, the imputed values from the imputation method on each new dataset after the (delete-1) jackknife are expected to be very similar to those from another new dataset where another sample is deleted. Therefore, even for the imputation method such that learning about the parameters in the imputation model involves both the reference panel and study individuals, repetition of the jackknife might not be necessary.

Second, in many situations the jackknife requires much fewer computations than the bootstrap. There is a substantial deduction in computations when the observed data are categorical with a finite number of possible values. Even for continuous data, when the number of observations is large enough, some observations share the same values, therefore for such observations the test statistic based on *n*−1 samples is the same. Thus, it is not necessary to compute the statistic as large as a sample size.

#### *Remark*.

We show the exact form of the jackknife variance estimator as follows. Let $\hat {\boldsymbol {\beta }}_{n}$ be the estimate of ***β*** and $\hat {\boldsymbol {\beta }}^{i}_{n-1}$ be estimate of *β* after *y*_*i*_ is deleted from the samples. Let $\hat {\boldsymbol {\beta }}^{i}_{\textit {ps}}= n\hat {\boldsymbol {\beta }}_{n}-(n-1)\hat {\boldsymbol {\beta }}^{i}_{n-1} $ be the pseudo sample value. Then the jackknife variance estimator for $\hat {\boldsymbol {\beta }}_{n}$ is $\frac {1}{n(n-1)}\sum _{i=1}^{n}\left (\hat {\boldsymbol {\beta }}^{i}_{\textit {ps}}-\tilde {\boldsymbol {\beta }}\right) \left (\hat {\boldsymbol {\beta }}^{i}_{\textit {ps}}-\tilde {\boldsymbol {\beta }}\right)^{T}$ where $\tilde {\boldsymbol {\beta }}=\frac {1}{n} \sum _{i=1}^{n} \hat {\boldsymbol {\beta }}^{i}_{\textit {ps}}$.

## Results

### Simulation setting

We performed simulation studies to explore the validity and power of WTT and WTT_*c*_ in realistic settings. We considered that the general population consists of a total of 10,000 individuals. The general population was used for assigning the genomic region of study subjects, not for being a reference in imputation of study subjects where study subject is one replica in the simulated dataset. We generated genotype data of the individuals in the general population based on the observed haplotype distributions in 180 kb and 174 kb regions of chromosomes 18 and 21 from the HapMap CEU samples using GWAsimulator [20], respectively. Thus, the simulated individuals have genotypes in the regions of chromosomes 18 and 21 with similar LD structure to that of the HapMap CEU samples. We chose a pair of SNPs in chromosomes 18 and 21 to be markers *G* and *H* which would be untyped in the simulated study data. For each study subject, one individual was randomly sampled from the general population conditioned on the genotypes at markers *G* and *H* for assigning the genotype data in the genomic region to the study subject (i.e., for each study subject, one individual whose genotype is the same as the study subject at SNP of interest (markers *G* or *H*) was sampled out of the general population and the whole genomic region of the sampled individual is assigned to the study subject.).

In each simulation for study data, we generated *n* joint genotypes at markers *G* and *H* using Bayes rule under the multiplicative penetrances model (i.e., the null model) and the two-locus models shown in Table 1. That is, we obtained the joint genotype frequencies for cases (i.e., subjects in study data), by using penetrance of the two-locus model and joint genotype frequency of markers *G* and *H* in the general population where markers *G* and *H* are in LE (i.e., the joint genotype frequency is the product of the marginal genotype frequencies). The marginal frequency of each marker was set as an estimator from the general population generated using GWAsimulator. See Tables S2 and S3 in Additional file 1 for all the marginal genotype frequencies and the values of Table 1 used in the simulation studies.

To quantify how much information for markers *G* and *H* is contained in the other SNPs, *M*_*D*_ [21], a measure for the amount of missing information with respect to multi-locus LD, was computed using the TUNA software [22] on the HapMap CEU samples for each marker. *M*_*D*_ measures the correlation between the imputed SNP and a set of typed SNPs and ranges from zero to one. To impute the genotypes at markers *G* and *H*, we chose a genotype imputation method, IMPUTE [12]. We performed marginal imputation with the reference as CEU HapMap Phase I and II (i.e., 120 haplotypes). Then we took a product of the marginally imputed genotype frequencies to obtain a joint genotype frequency for untyped markers *G* and *H* for each study subject.

In evaluating the tests for untyped markers, we assumed that genotypes at markers *G* and *H* were unobserved. The performances of Wald test (4) and ICPT were very similar to those of WTT and WTT_*c*_, respectively, on both imputed and genotyped data (results not shown). Therefore, we conducted the following three types of analysis : (A) perform WTT and WTT_*c*_ on dosage at untyped markers where *V*^0*u*^ was estimated by applying the jackknife on imputed values, (B) perform the Wald test (4) and ICPT on dosage at untyped markers, treating dosage as observed value, which are referred to as Dosage/WTT and Dosage/ WTT_*c*_, respectively, and (C) perform the Wald test (4) and ICPT on the actual genotypes at markers *G* and *H*, which are denoted as Truth/WTT and Truth/ WTT_*c*_, respectively. The dosage was obtained based on $\hat {\boldsymbol {p}}_{0}= (\hat {p}_{lm,0})_{l,m=0,1,2}$. Each analysis was performed on 5000 cases. For each model, 3000 and 100 studies were generated for evaluation of size and power, respectively. All the tests were evaluated at significance levels of 0.01 and 0.05 for size and power, respectively.

### Simulation results

Tables 2 and 3 present the type I error and the power with two pairs of SNPs which show different values of *M*_*D*_, leading to different levels of multi-locus LD. The results shown in Table 2 are for the case where *M*_*D*_= 0.9003 for marker *G* and *M*_*D*_= 0.7615 for marker *H*. Table 3 displays the results for a pair of SNPs where the values of *M*_*D*_ are 0.7266 and 0.7345 for markers *G* and *H*, respectively. Simulation results show that WTT and WTT_*c*_ have proper type I error under the level 0.01. We can see that Dosage/WTT and Dosage/ WTT_*c*_ are very conservative and are much less powerful than WTT and WTT_*c*_ across the disease models, respectively. WTT_*c*_ shows higher power than WTT, seen in the case when the corresponding tests were applied on the true genotypes.

**Table 2:** Size and power of tests for gene-gene interactions

Model	WTT	WTT_*c*_	Dosage/	Dosage/	Truth/	Truth/
			WTT	WTT_*c*_	WTT	WTT_*c*_
Null	0.007	0.007	0	0	0.008	0.008
D ∪D	0.55	0.58	0.15	0.28	0.81	0.87
R ∪R	0.57	0.61	0.30	0.36	0.81	0.83
D ∪R	0.42	0.46	0.13	0.17	0.68	0.73
R ∩D	0.22	0.26	0.05	0.08	0.40	0.46
R ∩R	0.67	0.69	0.34	0.42	0.85	0.90
D ∩D	0.15	0.19	0.01	0.04	0.27	0.32

*M*_*D*_ for markers *G* and *H* using the HapMap CEU samples are 0.9003 and 0.7615, respectively. WTT and WTT_*c*_ correspond to tests (5) and (6) where ***V*** ^0*u*^ was estimated by applying the jackknife on imputed values. Dosage/WTT and Truth/WTT are test (4) using the asymptotic variance based on expected genotypes and true genotypes, respectively. Dosage/ WTT_*c*_ and Truth/ WTT_*c*_ are ICPT on expected genotypes and true genotypes, respectively. Models are described in Table 1. Number of individuals in cases is 5000. Significance levels for size and power are 0.01 and 0.05, respectively. WTT, Wald-type test; WTT_*c*_, Wald-type test with constraints

**Table 3:** Size and power of tests for gene-gene interactions

Model	WTT	WTT_*c*_	Dosage/	Dosage/	Truth/	Truth/
			WTT	WTT_*c*_	WTT	WTT_*c*_
Null	0.011	0.010	0	0	0.006	0.008
D ∪D	0.14	0.16	0	0.04	0.56	0.60
R ∪R	0.17	0.21	0.02	0.03	0.86	0.94
D ∪R	0.15	0.17	0.04	0.08	0.70	0.77
R ∩D	0.15	0.17	0.02	0.03	0.71	0.77
R ∩R	0.22	0.25	0.03	0.04	0.81	0.84
D ∩D	0.13	0.14	0.01	0.04	0.73	0.76

*M*_*D*_ for markers *G* and *H* using the HapMap CEU samples are 0.7266 and 0.7345, respectively. WTT and WTT_*c*_ correspond to tests (5) and (6) where ***V*** ^0*u*^ was estimated by applying the jackknife on imputed values. Dosage/WTT and Truth/WTT are test (4) using the asymptotic variance based on expected genotypes and true genotypes, respectively. Dosage/ WTT_*c*_ and Truth/ WTT_*c*_ are ICPT on expected genotypes and true genotypes, respectively. Models are described in Table 1. Number of individuals in cases is 5000. Significance levels for size and power are 0.01 and 0.05, respectively. WTT, Wald-type test; WTT_*c*_ test, Wald-type test with constraints

Overall, the relative powers of WTT and WTT_*c*_ compared with Truth/WTT and Truth/ WTT_*c*_, ideal tests which were performed on the true genotypes at markers *G* and *H*, are lower as *M*_*D*_ decreases for markers *G* and *H*, respectively (see Tables 2 and 3). This is generally expected because of lower information content.

We conducted additional simulation studies to investigate the validity of the proposed tests with various values of *M*_*D*_ by generating study subjects under the null model (i.e., multiplicative penetrances model). We chose different markers so that we could change the values of *M*_*D*_ for marker *G* while fixing the value of *M*_*D*_ at marker *H* as 0.7615. As shown in Table 4, simulation studies suggest that the type I errors for WTT and WTT_*c*_ on the imputed genotypes and the Wald test (4) and ICPT on the true genotypes are well controlled. Though the amount of information in observed genotypes that can predict the untyped SNPs is very low (e.g., *M*_*D*_= 0.0235), still the tests maintain their type I errors around the nominal value of 1 *%*. This is due to the fact that Wald-type test is valid as long as the estimator for the variance is consistent. Since the jackknife can produce the consistent variance estimator, the validity of the proposed tests is robust to the level of multi-locus disequilibrium.

**Table 4:** Size of tests for gene-gene interactions under the various values of *M*_*D*_

*M*_*D*_ of marker *G*	WTT	WTT_*c*_	Truth/ WTT	Truth/ WTT_*c*_
0.0235	0.008	0.006	0.006	0.006
0.2222	0.016	0.008	0.010	0.009
0.3528	0.011	0.006	0.007	0.005
0.6057	0.011	0.006	0.010	0.008
1	0.007	0.004	0.010	0.008

WTT corresponds to test (5). WTT_*c*_ corresponds to test (6). Truth/WTT and Truth/ WTT_*c*_ are test (4) and ICPT on true genotypes, respectively. The values of *M*_*D*_ for marker *G* range from 0.0235 to 1 while the value of *M*_*D*_ for marker *H* is fixed as 0.7615. Number of individuals in cases is 5000. Significance level =0.01. WTT, Wald-type test; WTT_*c*_, Wald-type test with constraints

### Environment and genetics in lung cancer etiology (eagle) study

We applied for the proposed methodology to a dataset from a population-based case-control study on lung cancer, the Environment and Genetics in Lung Cancer Etiology (EAGLE), which enrolled lung cancer cases and controls in Italy between 2002 and 2005. The study protocol has been described in Landi *et al.* [23]. The study has received approval from the Institutional Review Board of the University of Milan and of the National Cancer Institute. All participants signed a written informed consent. Here, we focus on data involving 1917 cases after exclusion of individuals because of informed consent issues and for quality control reasons. rs8034191 which is located on chromosome 15q25.1 has been reported as a signal associated with lung cancer [24, 25]. Also, genetic variants in TERT and CLPTM1L on chromosome 5p15.33 have been shown to be associated with risk of lung cancer [26, 27]. In the EAGLE dataset, rs8034191 is typed completely with no missing. The untyped SNPs are imputed using IMPUTE2 with CEU HapMap Phase III and 1000 Genomes Project data [28] as the reference. We performed SNP-SNP interaction testing, WTT_*c*_, on lung cancer between rs8034191 and SNPs which are typed and untyped on a 1.8Mb region where both TERT and CLPTM1L are located. We analyzed the typed SNPs such that minor allele frequency (MAF) ≥ 0.05 without any missing, which led to 219 SNPs in the region. The untyped SNPs in the analysis are the 4573 SNPs on the region containing TERT and CLPTM1L which meet the following criteria : (1) expected MAF ≥ 0.05 and (2) information measure provided by IMPUTE2 ≥ 0.5.

**Fig. 1:**
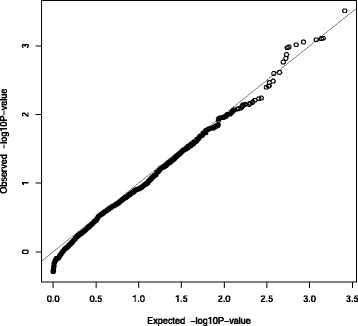
Q-Q plot for WTT_*c*_ for interactions on lung cancer. *p*-values are from WTT_*c*_ for interactions between rs8034191 and untyped SNPs on regions where both TERT and CLPTM1L are located. Q-Q, quantile-quantile; WTT_*c*_, Wald-type test with constraints

As shown in Fig. 1, quantile-quantile (Q-Q) plot of the *p*-value distribution from WTT_*c*_ on untyped SNPs shows that the deviations of the *p*-values from the Uniform distribution are minor except in the extreme tails, which is expected in GWAS where most of *p*-values are expected to follow the Uniform distribution.

**Fig. 2:**
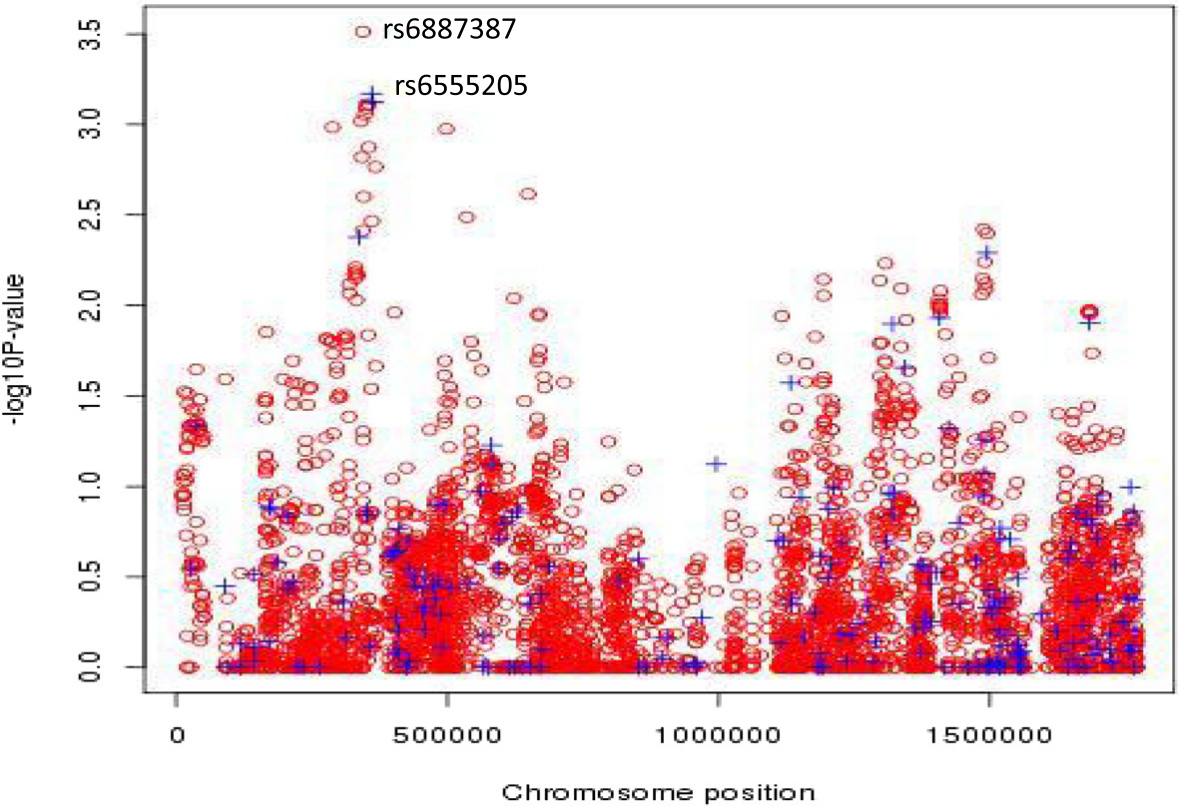
Results of WTT_*c*_ for interactions on lung cancer for the region of TERT and CLPTM1L. − log10*p*-value is plotted against chromosomal location for the typed and untyped SNPs in blue (+) and red (o), respectively. WTT_*c*_, Wald-type test with constraints

We show the results of WTT_*c*_ for interaction in the 1.8Mb region in Fig. 2, where we plot − log10 *p*-value for typed SNPs and untyped SNPs. The untyped SNPs show very similar pattern to the typed SNPs and detailed results for the region. The typed SNPs as local maxima in − log10*p*-value are flanked by a column of other untyped SNPs, which is a pattern characteristic of true signal rather than genotyping error (If it is a true signal rather than artifacts such as genotyping error, SNPs in LD tend to show similar level of signal). Therefore, the analysis on untyped SNPs allows us to have much more comprehensive view. We can see that basically all of the typed SNPs which show local peak tend to have another nearby untyped SNP which shows slightly enhanced interaction signals. For example, untyped SNP rs6887387 located at 344218 with an information metric as 0.948 computed by IMPUTE2 shows stronger evidence against no interaction than typed SNP rs6555205 located at 360543.

## Discussion

We have presented new tests for gene-gene interaction on marginally imputed values from the existing imputation methods at untyped SNPs (e.g., IMPUTE/IMPUTE2, MACH [8], BEAGLE [11], and PLINK [13]). Our methods, WTT and WTT_*c*_, are constructed under the null hypothesis in the Wald-like form where we obtain the estimator of the variance (under the null) which directly takes into account uncertainty of inferring genotypes at the untyped SNPs using the jackknife. For detecting interactions among SNPs with small effects, larger number of subjects are needed for a discovery and it has been shown that a meta analysis could be statistically as efficient as an analysis by pooling individual-level data [5]. Proposed interaction analysis of untyped SNPs can permit such a meta-analysis with different genotyping platforms, which will lead to an improvement in power. Even though in Methods section we have presented the proposed tests for the untyped SNPs, an extreme form of missing genotype, both WTT and WTT_*c*_ are also applicable when each marker is missing completely at random or markers are jointly missing completely at random (see Additional file 1).

For testing (pure) interaction which is defined as deviation from multiplicative penetrances, case-only analysis could be much more powerful than logistic regression method using cases and controls [16]. For case-only studies, one could develop a test statistic based on genotype distribution directly conditioned on cases. However, when the test statistics are constructed on the distribution of genotypes and unmeasured genotypes are simply replaced as dosage which is a popular approach as downstream analysis after the imputation, the tests can suffer from the loss of the power due to overestimation of variance. When we compared the average of the variance estimator $\hat {\boldsymbol {V}}^{0u}$ across simulations with the empirical variance estimator of $\hat {\boldsymbol {\beta }}^{0u}$ across simulations, we observed that the variance estimator of $\hat {\boldsymbol {\beta }}^{0u}$ from the case where one just simply replaces unobserved genotype as dosage is overestimated (i.e., $\frac {1}{K} \sum _{k=1}^{K} \hat {V}_{k,jj}^{0u} > \frac {1}{K-1} \sum _{k=1}^{K} \left (\hat {\beta }_{k,j}^{0u}-\frac {1}{K}\sum _{k=1}^{K} \hat {\beta }_{k,j}^{0u}\right)^{2}$ for *j*=1,…,4 where *K* is the number of simulations, and $\hat {V}_{k,jj}^{0u}$ and $\hat {\beta }_{k,j}^{0u}$ are the (*j*,*j*)th diagonal values of $\hat {\boldsymbol {V}}^{0u}$ and the *j*th element of $\hat {\boldsymbol {\beta }}^{0u}$ obtained from simulated data *k*, respectively (see Additional file 1: Table S1). With increasing imputation uncertainty, the imputed dosage becomes less variable due to lack of information. Thus the variance of the dosage would be smaller than the variance of the observed genotype, which will lead to overestimation of the variance when dosage is treated as observed data. The loss in power due to overestimation of the variance will be even worse in meta-analyses since such overestimation of variance will be cumulative when we combine interaction evidence across studies.

In the simulation studies, we used the significance level of 0.01 to validate the test statistics. The simulation studies showed that the type I error rates of proposed statistics were close to the nominal significance level 0.01. To see whether the tests could have size matching reasonably well with the other nominal significance levels, we performed a Kolmogorov-Smirnov (KS) test comparing the distribution of the *p*-values from the null scenario with the Uniform(0,1) distribution. The KS test comparing the distribution of *p*-values with the Uniform distribution is a test whether the null distribution is accurate, which is a stronger condition than maintaining a nominal size of test. WTT follows the correct null distribution (results not shown). Thus, we found that the asymptotic distribution of WTT is accurate, indicating that at any significance level, it is expected that the proper type I error is achieved around the nominal type I error. WTT_*c*_ is slightly conservative based on the KS test (i.e., the null p-value from those tests is stochastically greater than the Uniform(0,1) random variable).

We investigated the effect of multi-locus LD on the proposed tests. As expected, power decreases as multi-locus LD information among SNPs becomes lower. However, with respect to the validity, simulation studies show that WTT and WTT_*c*_ are robust to the amount of information which could be used to predict the genotypes at the untyped SNP. This is due to the fact that the jackknife provides the consistent variance estimator. With the similar reason, when the reference set is a poor representation of study dataset or the reference set is small so that $\hat {\boldsymbol {\eta }}_{0}$ has a big variance, the tests would still be valid since the jackknife could take into account the poor estimation of ***η***^0*u*^. In other words, the jackknife properly accounts for the additional variability from estimation of ***η***^0*u*^ when the jackknife estimates the variance of $\hat {\boldsymbol {\beta }}^{0u}$. It is worth noting that in our simulations, we used only 60 HapMap CEU samples for imputation and WTT and WTT_*c*_ are valid and significantly more powerful than the tests which simply replace unmeasured genotypes as dosage.

To evaluate the performance of WTT_*c*_ over WTT, we compared the power of the two tests. We showed that consistent improvement in performance of WTT_*c*_ could be obtained by restricting the parameter space under the alternative hypothesis. With the same argument as in the typed markers [16], we expect that the gains of using WTT_*c*_ will grow with the type I error, although we have not studied this. However, WTT for interaction between untyped SNPs is very simple and computationally tractable so it has its own advantage compared with WTT_*c*_.

In this paper, we developed interaction testing approaches by considering SNP level. We note that several methods to perform interaction analysis at the gene level have been proposed [29, 30]. Working at the gene-level is computationally feasible and reduces the burden of multiple-testing correction. However, SNP-based interaction analysis can perform well when a single SNP in a gene interacts with a single SNP in another gene [29, 30]. One needs to bear in mind the trade-offs between the interaction analysis for SNP and gene levels.

Even though the jackknife is a great method to estimate the variance which could take into account the uncertainty at untyped SNPs, it may not perform well when the allele frequency is low. However, this is not just the problem of the jackknife. The test based on the asymptotic theory is not suitable for low allele frequencies. For rare SNPs, one way to solve the problem is that we can combine several genotype configurations although some information might be lost.

We believe that two-stage strategies, in which interaction test is performed only on a subset of markers based on marginal associations, are more appropriate than exhaustive search for identifying gene-gene interactions, in terms of computation and a penalty for multiple testing. Under the strategy, the proposed tests are computationally feasible.

## Conclusions

The proposed methodologies provide a promising solution for the gene-gene interaction tests using imputed values from external imputation algorithms in the two-stage strategy. We are currently extending our approach to the case where SNPs are in LD, which involves controls as well as cases.

## Additional file

Additional file 1 **Supplementary Material.** 1. the form of the weights in the chi-bar distribution of the WTTc,. 2. how the proposed methods are applicable for genotype at each marker missing completely at random or genotypes at two markers jointly missing completely at random. 3. the simulation result comparing the average of variance estimator with the empirical variance estimator when dosage was treated as observed genotype data (Table S1). 4. the values used in simulation studies (Tables S2 and S3).
