# Supplementary material for: Jackknife-based gene-gene interactiontests for untyped SNPs
Source: BMC Genet. 2015 Jul 18;16:85. doi: 10.1186/s12863-015-0225-9 (PMC4506584; doi:10.1186/s12863-015-0225-9)
Supplement: Supplementary file 1 — Supplementary Material. 1. the form of the weights in the chi-bar distribution of the WTTc,. 2. how the proposed methods are applicable for genotype at each marker missing completely at random or genotypes at two markers jointly missing completely at random. 3. the simulation result comparing the average of variance estimator with the empirical variance estimator when dosage was treated as observed genotype data (Table S1). 4. the values used in simulation studies (Tables S2 and S3). [file 12863_2015_225_MOESM1_ESM.pdf]

Supplementary Material to

Jackknife-based gene-gene interaction tests for untyped SNPs

Minsun Song

Division of Cancer Epidemiology and Genetics, National Cancer Institute, National Institutes of Health, Rockville, Maryland 20850

1 WEIGHTS IN THE CHI-BAR-DISTRIBUTION

In this section, we show the formulas for the weights w_l, l=1,2,3,4 of a chi-bar distribution (i.e., weighted sum of χ^2 distributions), the asymptotic distribution of WTT$_c$. In the case of ICPT for typed SNPs, weights are derived from an asymptotic covariance, which is a function of joint genotype frequencies for cases.[1] However, for untyped SNPs, obtaining weights based on an analytical form of covariance estimator for typed SNPs using the estimated joint genotype frequencies (i.e., $E[N_{l,m}|y_{\text{obs}}, \hat{\boldsymbol{\eta}}_0]/n$) is not appropriate, since the uncertainty due to ungenotyped SNPs is not properly taken into account. Instead, using the jackknife, we can obtain the estimator of V^{0u}, variance of $\hat{\boldsymbol{\beta}}^{0u}$. We denote ρ_{ij} be the (i,j)th element of $(\text{diag}(V^{0u}))^{-1/2}V^{0u}(\text{diag}(V^{0u}))^{-1/2}$. Then the weights are given by Kudo[2] and Shapiro,[3]

$$
\begin{aligned}
w_1 &= \frac{1}{8}\pi^{-1}(8\pi - \sum_{i>j;i,j\neq k} \cos^{-1}\rho_{ij.k}),\\
w_2 &= \frac{1}{4}\pi^{-2} \sum_{i>j,k>l;k,l\neq i,j} (\cos^{-1}\rho_{ij})(\pi - \cos^{-1}\rho_{kl.ij}),\\
w_3 &= \frac{1}{8}\pi^{-1}(-4\pi + \sum_{i>j;i,j\neq k} \cos^{-1}\rho_{ij.k}),\\
w_4 &\leq \min(1 - w_1 - w_2 - w_3, \min_{(i,j,k)\subseteq(1,2,3,4)} \frac{1}{4\pi} \times (2\pi - \cos^{-1}\rho_{ij} - \cos^{-1}\rho_{ik} - \cos^{-1}\rho_{jk})),
\end{aligned}
$$

where $\hat{\beta}_j^{0u}$ is the jth element of $\hat{\boldsymbol{\beta}}^{0u}$ for j =1,2,3,4 and $\rho_{ij.k}$ and $\rho_{kl.ij}$ are conditional correlation between $\hat{\beta}_i^{0u}$ and $\hat{\beta}_j^{0u}$ given $\hat{\beta}_k^{0u}$ and the conditional correlation between $\hat{\beta}_k^{0u}$ and $\hat{\beta}_l^{0u}$

given $\hat{\beta}_i^{0u}$ and $\hat{\beta}_j^{0u}$, respectively.

2 MISSING GENOTYPE DATA

When genotype at each marker is missing completely at random or genotypes at two markers are jointly missing completely at random, both of WTT and WTT$_c$ for interactions are applicable. For example, for genotypes at two markers jointly missing completely at random, $E[N_{l,m}|y_{\text{obs}}, \hat{\boldsymbol{\eta}}_0] = \sum_{i=1}^{n} \hat{p}_{i,lm,0}^{*,G,H}$ where

$$\hat{p}_{i,lm,0}^{*,G,H} = \delta_i I(y_{i,G} = l, y_{i,H} = m) + (1 - \delta_i)\hat{p}_{i,lm,0}^{G,H},$$

and $\delta_i = 1$ if both $y_{i,G}$ and $y_{i,H}$ are observed and δ_i=0 otherwise.

3 VARIANCE OF DOSAGE

For the case where dosage was treated as observed genotype data, we compared the average of the variance estimator $\hat{\boldsymbol{V}}^{0u}$ across K simulations with the empirical variance estimator of $\hat{\boldsymbol{\beta}}^{0u}$ across K simulations under the null scenario presented in Table 2 of the main text. $K = 3000$. For $j = 1, ..., 4$, $\hat{V}_{k,jj}^{0u}$ and $\hat{\beta}_{k,j}^{0u}$ are the (j, j)th diagonal values of $\hat{\boldsymbol{V}}^{0u}$ and the jth element of $\hat{\boldsymbol{\beta}}^{0u}$ obtained from simulated data k, respectively, where $k = 1, ..., 3000$. Rows (A) and (B) present $\frac{1}{K}\sum_{k=1}^{K} \hat{V}_{k,jj}^{0u}$ and $\frac{1}{K-1}\sum_{k=1}^{K}(\hat{\beta}_{k,j}^{0u} - \frac{1}{K}\sum_{k=1}^{K} \hat{\beta}_{k,j}^{0u})^2$, respectively. We can observe that for each j, the average of the variance estimator $\hat{V}_{jj}^{0u}$ across K simulations is larger than the empirical variance estimator of $\hat{\beta}_j^{0u}$ across K simulations.

Table S1: **Comparison between average of the variance estimator and empirical variance estimator.** β_{lm} for $l, m = 0, 1$ correspond to (1). Rows (A) and (B) present $\frac{1}{K}\sum_{k=1}^{K} \hat{V}_{k,jj}^{0u}$ and $\frac{1}{K-1}\sum_{k=1}^{K}(\hat{\beta}_{k,j}^{0u} - \frac{1}{K}\sum_{k=1}^{K}\hat{\beta}_{k,j}^{0u})^2$, respectively. $K = 3000$.

	β_{00}	β_{01}	β_{10}	β_{11}
(A)	0.014	0.014	0.007	0.007
(B)	0.007	0.007	0.004	0.004

4 SIMULATION SETTING

Table S2: **Genotype frequencies for the scenarios used in simulation studies.** For Tables 2 and 3, marginal genotype frequencies are fixed across all 7 scenarios.

Genotype frequencies for SNPs G and H	aa	Aa	AA	bb	Bb	BB
Table 2	0.175	0.484	0.341	0.118	0.457	0.426
Table 3	0.435	0.447	0.119	0.391	0.474	0.135
Table 4 (M_D =0.0235)	0.280	0.500	0.220	0.118	0.457	0.426
Table 4 (M_D =0.2222)	0.363	0.477	0.160	0.118	0.457	0.426
Table 4 (M_D =0.3528)	0.097	0.434	0.469	0.118	0.457	0.426
Table 4 (M_D =0.6057)	0.138	0.473	0.389	0.118	0.457	0.426
Table 4 ($M_D = 1$)	0.463	0.438	0.099	0.118	0.457	0.426

Table S3: **Values of g and f for the scenarios used in simulation studies to evaluate power.**

	Table 2		Table 3	
	g	f	g	f
D∪D	0.05	0.08	0.05	0.06
R∪R	0.08	0.10	0.04	0.06
D∪R	0.07	0.09	0.03	0.04
R∩D	0.08	0.10	0.03	0.04
R∩R	0.08	0.10	0.04	0.06
D∩D	0.08	0.10	0.05	0.06

References

[1] Song M and Nicolae DL. Restricted parameter space models for testing gene-gene interaction. *Genetic Epidemiology*, 33(5):386–393, 2009.

[2] Kudo A. A multivariate analogue of the one-sided test. *Biometrika*, 50:403–418, 1963.

[3] Shapiro A. Asymptotic distribution of test statistics in the analysis of moment structures under inequality constraints. *Biometrika*, 72(1):133–144, 1985.
